# A new pathway for considering trigger factors based on parallel-serial connection models and displaying the relationships of causal factors in low-probability events

**DOI:** 10.1186/s12874-023-01919-3

**Published:** 2023-04-15

**Authors:** Liu Hui

**Affiliations:** College of Medical Laboratory, Dalian Medical University, Dalian, 116044 China

**Keywords:** Case–control studies, Cohort studies, Data visualisation, Effect size, Multivariate models

## Abstract

**Background:**

To determine the effect size of observed factors considering trigger factors based on parallel-serial models and to explore how multiple factors can be related to the result of complex events for low-probability events with binary outcomes.

**Methods:**

A low-probability event with a true binary outcome can be explained by a trigger factor. The models were based on the parallel-serial connection of switches; causal factors, including trigger factors, were simplified as switches. Effect size values of an observed factor for an outcome were calculated as SAR = (Pe-Pn)/(Pe + Pn), where Pe and Pn represent percentages in the exposed and nonexposed groups, respectively, and SAR represents standardized absolute risk. The influence of trigger factors is eliminated by SAR. Actual data were collected to obtain a deeper understanding of the system.

**Results:**

SAR values of < 0.25, 0.25–0.50, and > 0.50 indicate low, medium, and high effect sizes, respectively. The system of data visualization based on the parallel-serial connection model revealed that at least 7 predictors with SAR > 0.50, including a trigger factor, were needed to predict schizophrenia**.** The SAR of the HLADQB1*03 gene was 0.22 for schizophrenia.

**Conclusions:**

It is likely that the trigger factors and observed factors had a cumulative effect, as indicated by the parallel-serial connection model for binary outcomes. SAR may allow better evaluation of the effect size of a factor in complex events by eliminating the influence of trigger factors. The efficiency and efficacy of observational research could be increased if we are able to clarify how multiple factors can be related to a result in a pragmatic manner.

## Background

A complex event, such as success in a certain field, longevity or the occurence of disease, is the result of multifactor interactions [1–3]. Such events are associated with multiple factors and do not depend on a specific factor. The association between multiple factors and results is usually explained by a superimposed model, in which different risk factors play a role in an additive manner that leads to a continuous outcome. Although this continuous outcome can be separated into two states by a threshold, it is not a true binary outcome; thus, it is considered a transformed binary outcome. For low-probability events with true binary outcomes, the superimposed model may not be reasonable, which makes observations and research of such events difficult. Therefore, it is important to distinguish among continuous outcomes, transformed binary outcomes, and true binary outcomes and to establish relevant models to solve the problem.

A trigger factor is defined as a decisive factor in the outcome. In other words, the outcome would not have occurred without the trigger factor, although a sufficient number of relevant factors exist to influence the outcome. In general, the frequency of such trigger factors is very low; hence, trigger factors are difficult to detect. A low-probability event with a true binary outcome can be explained by a trigger model, in which the trigger factor plays a key role that leads to true binary outcomes. However, the models used to assess the associations between multiple factors and binary outcomes do not take trigger factors into account.

Cohort studies are used to examine correlations between factors and diseases. In cohort studies, a suspected risk factor is the exposure factor, and exposed and unexposed subjects are observed until they develop the outcome of interest. However, long-term observations of large populations are necessary for research on low-incidence diseases, which increases the study’s costs and duration as well as loss to follow-up, thereby precluding the use of cohort studies to investigate many diseases; therefore, cohort design is not often the practical choice. Hence, a new model of parallel and serial connection switches is proposed that takes both trigger factors and the effect size of the observed factor into account.

**Fig. 1:**
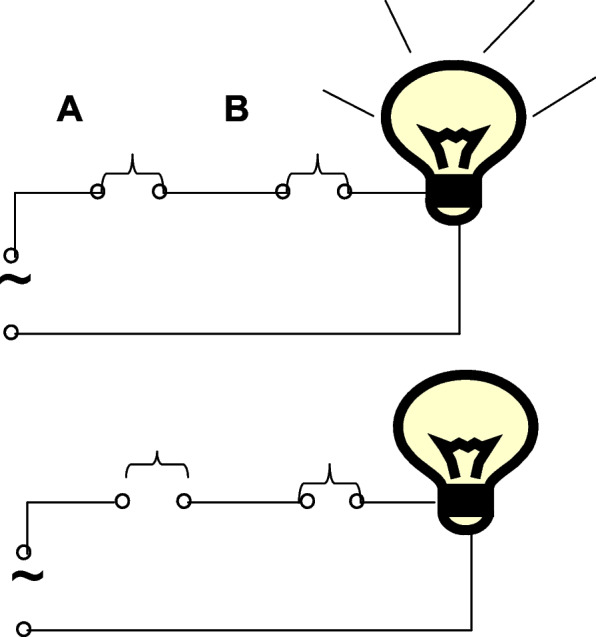
Model of a serial connection

**Fig. 2:**
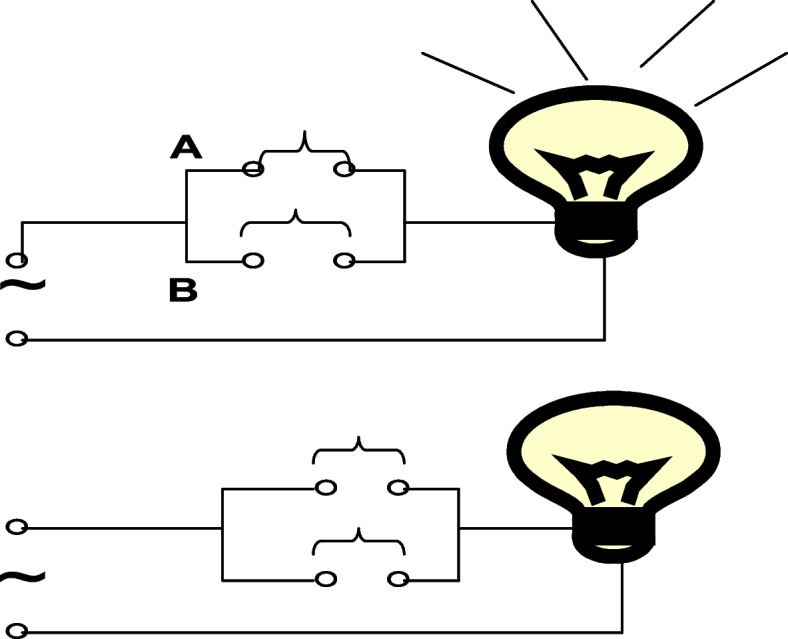
Model of a parallel connection

A serial connection model yields a result only when all the related factors are present (Fig. 1); in contrast, a parallel connection model yields results if any of the related factors are present (Fig. 2). The three models (serial connection, parallel connection, and superimposed models) may be used to model complex events influenced by multiple synergetic factors; the superimposed model yields obtain continuous outcomes, while the remaining two models may explain an event with true binary outcomes accurately. This is, to our knowledge, the first report to present inferece about the role of a complex event-related factor using a switch connection model.

Data visualization entails the visual representation of data to communicate information effectively through graphical means; it can clearly display fuzzy relationships among causal factors in a complex event [4–6]. Information visualization is generally accepted as a computerized method that involves selecting, transforming, and representing data in such a way (commonly in a graphical manner) that the information can be identified by sensory organs. Although researchers and practitioners often create patterns that can be visually identified (e.g., charts, graphs, and interactive displays) to solve a large range of problems, there are no definite, accepted methods to identify these complex relationships, especially complex events involving multiple causes (i.e., "multicause and one-effect" events). The present study explored the association between the cause and the result of a complex event based on parallel and serial connections, and it established a model of associations for multivariate data visualization of a complex event to identify the appropriate model to use for drawing inferences about low-probability events with binary outcomes. This modelling approach is valuable because many important events have a low probability of occurrence in the population, such as the occurrence of disease. The purpose of this study was to obtain a deeper understanding of the regularity of the occurrence of complex events and to explore new pathways to study low-probability events.

## Methods

### Analysing the interaction pattern of factors

**Fig. 3:**
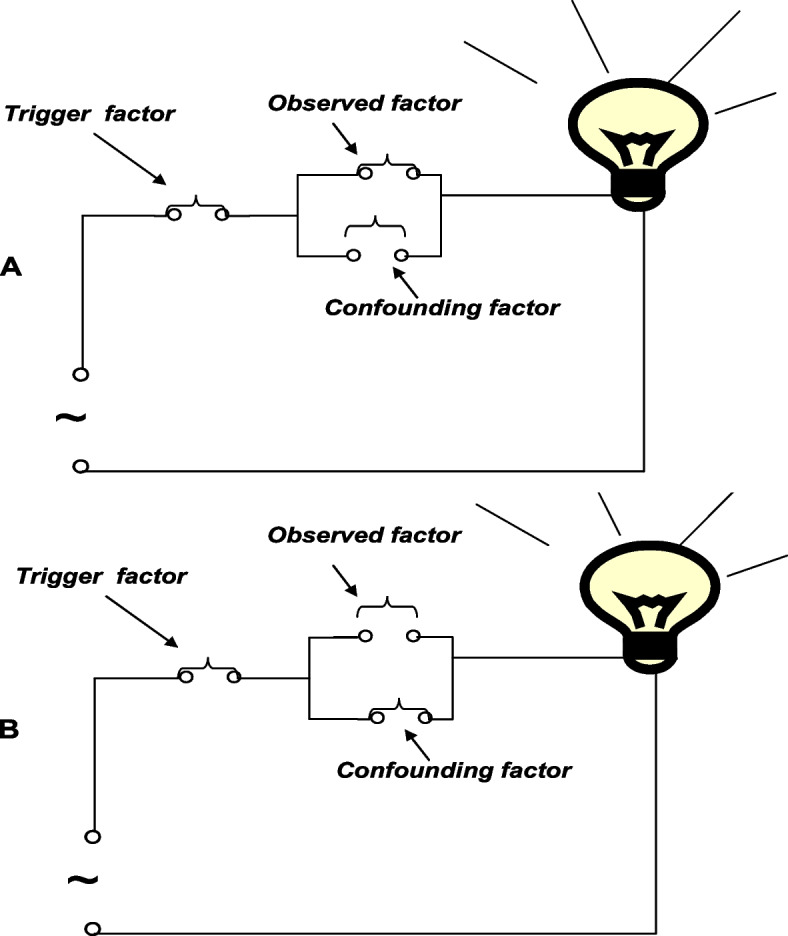
The parallel and serial-connection model of a complex event with an observed factor, a trigger factor and a confounding factor. **A** pattern for exposed group; **B** pattern for non-exposed group. The expectation that the incidence in the exposure group may be not 1: (**A**) due to an observed factor acting with a trigger factor; and (**B**) the expectation that the incidence in the non-exposure group may be not zero due to confounding factors acting with a trigger factor

The incidence of a binary outcome can be considered as resulting from the accumulation of risk factors according to the models of parallel and serial-connection switches, as shown in Figs. 1 and 2. According to these models, a parallel-connection risk pattern means that the incidence of disease should be 1.0 in the exposure group of a cohort study. However, high incidence rates (up to 1.0) in the exposure group are not frequently observed for low-probability events. It is assumed that the serial-connection risk factors, including trigger factors, play a role in this difference, as shown in Fig. 3.

The expectation that the incidence of an event in the exposure group is less than 1 can be illustrated by showing that an observed factor is influenced by other factors, including the trigger factor, according to a serial-connection model, as shown in Fig. 3A. Because the frequency of trigger factors is usually very low, a very high frequency of occurrence is not observed in the exposure group. Moreover, the expectation that the incidence in the nonexposure group is not zero can be illustrated by showing that some parallel-connection risk factors (confounding factors) influence the trigger factor, as shown in Fig. 3B; the larger the effect sizes of the observed factor are, the weaker the confounding factor.

### Calculating the effect sizes of a factor on the result

The first principle of this study is that the influence of the observed factors on the results is explained by the elimination of trigger factors. Absolute risk (AR, calculated as intergroup differences in the incidence in cohort studies) represents the mean probability of incidence due to an observed factor. If the sum of the percentage in the exposed group and the nonexposed group is one, the standardized AR (SAR) is calculated as follows:

1 $$SAR= \frac{TF \left(Pe-Pn\right)}{TF \left(Pe+Pn\right)}= \frac{\left(Pe-Pn\right)}{\left(Pe+Pn\right)}$$

where TF represents the trigger factor; Pn represents the observed percentage in the nonexposed group; and Pe represents the observed percentage in the exposed group. The influence of the trigger factors is eliminated by SAR. The range of SAR values is (0 ~ 1). SAR values of < 0.25, 0.25–0.50, and > 0.50 indicate low, medium, and high SAR, respectively. According to the parallel-connection model, SAR values over 0.50 indicate high-level intensity factors and are effective predictors. SAR is considered a trigger factor when it is close to 1.0.

### Displaying the relationship of causal factors in complex events

**Fig. 4:**
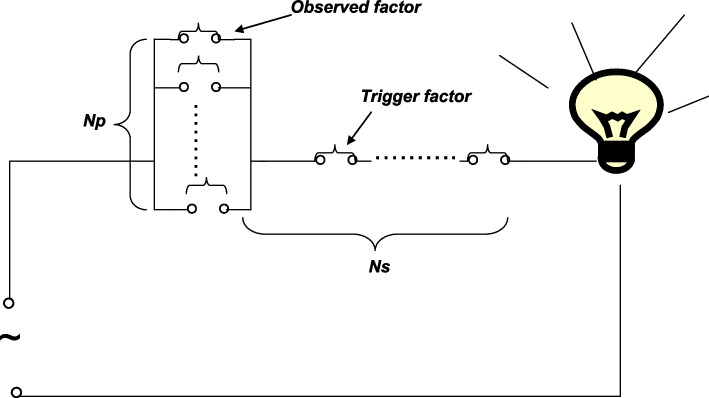
Displaying the relationship and strength of causal factors based on the parallel and serial connection of switches

The switch-on state was defined as the presence of the observed factor; thus, data visualization using switch-on, switch-off and the number of switches revealed patterns and levels of causal factors, as shown in Fig. 4. The models were based on parallel and serial connections of switches representing risk factors.

To displaying causal factors of low-probability events, the model assumes that risk factors are accumulated by a serial connection for lower probability events, as shown in Fig. 4. The serial model was based on the following assumptions: (1) the outcome is absent in the presence of a single causative factor; (2) the presence of each causative factor is independent of the other factors; and (3) a binary outcome occurs only if all the causative factors, including trigger factors, are present. Based on these assumptions, the number of predictive factors based on the serial model (Ns) can be determined by morbidity in the total population (m) as follows:

The frequency of a closed state was set to 0.5, 0.5^Ns^ = m.

Thus,

2 $$Ns=\frac{\log\left(m\right)}{\log\;0.5}$$

To display SAR, the model assumes that observed factors are accumulated by a parallel connection, as shown in Fig. 4. The parallel model was based on the assumption that the outcome would occur if any of the causative factors were present and that the presence of different causative factors would be independent of each other for a binary outcome regardless of factor superimposition.

The switch-on was set to the presence of causative factors; the observed factor and some confounding factors acted in the parallel model, as shown in Fig. 4. The number of observed factors based on the parallel model (Np) can be determined using SAR by setting the switches to an open state; the outcome will be present if any of the switches is closed (only one is closed at the same time).

3 $$Np=\frac{1}{SAR}$$

Thus, the larger the SAR is, the lower the number of confounding factors. For a given factor, if SAR > 0.50 and Np < 2.0, it can be considered a predictive factor. It can be considered a trigger factor when the SAR or Np values are close to 1.0.

### Actual data of a complex event for relationship visualization

Two different sets of data were used to evaluate effect sizes in complex events and used to provide a concrete example of information visualization. The first analysis assessed the role of a putative genetic factor in a complex disease, and the second assessed the role of the putative factor age in death.

Schizophrenia (SCH) is a polygenic disease that affects approximately 1% of the population [7]. It mostly develops after the age of 20–30 years and has a low incidence in older individuals [7–9]. Compared to other complex diseases, SCH is not significantly influenced by ageing, the environment, or diet. Genetic factors appear to play an important role in SCH [10]. Therefore, SCH can serve as a model for the evaluation of polygenes in complex diseases. Our previous work, which used a case‒control design, found that the frequencies of HLA-DQB1*03 were 0.533 in the SCH group and 0.420 in the control group (*p* = 0.035) [10], which could be used as an example of information visualization.

Regarding the relationship between age and death (in which the incidence is observed to increase with age), age can be considered to be an important factor in death. Therefore, age was chosen as an example to describe the role of ageing in death. Raw data were obtained from the literature [11]. These data were obtained from over 73 million people living in China.

## Results

### SAR, AR, RR and OR calculation

A standardized model with a special dataset (sum of incidence in two groups is 1.0) is presented in Table 1. The observed data with a lower incidence are due to the role of trigger factors in the original data. The simulation model also shows the relationships among SAR, AR and relative risk (RR; ratio of the incidences between the two groups of cohort studies). To understand the relationships among these variables, odds ratios (OR) were derived from case control studies.

**Table 1:** The special dataset based on the standardized model used to evaluate the relationships considering trigger factors among AR, RR and SAR

Original data			Observed data (trigger factors = 1%)			SAR	RR	SAR'
Exposure	Non-exposure	AR	Exposure	Non-exposure	AR			
0.55	0.45	**0.10**	0.0055	0.0045	0.0010	**0.10**	1.22	**0.10**
0.60	0.40	**0.20**	0.0060	0.0040	0.0020	**0.20**	1.50	**0.20**
0.65	0.35	**0.30**	0.0065	0.0035	0.0030	**0.30**	1.86	**0.30**
0.67	0.33	**0.33**	0.0067	0.0033	0.0033	**0.33**	2.00	**0.33**
0.70	0.30	**0.40**	0.0070	0.0030	0.0040	**0.40**	2.33	**0.40**
0.75	0.25	**0.50**	0.0075	0.0025	0.0050	**0.50**	3.00	**0.50**
0.80	0.20	**0.60**	0.0080	0.0020	0.0060	**0.60**	4.00	**0.60**
0.83	0.17	**0.67**	0.0083	0.0017	0.0067	**0.67**	5.00	**0.67**
0.85	0.15	**0.70**	0.0085	0.0015	0.0070	**0.70**	5.67	**0.70**
0.86	0.14	**0.71**	0.0086	0.0014	0.0071	**0.71**	6.00	**0.71**
0.88	0.13	**0.75**	0.0088	0.0013	0.0075	**0.75**	7.00	**0.75**
0.89	0.11	**0.78**	0.0089	0.0011	0.0078	**0.78**	8.00	**0.78**
0.90	0.10	**0.80**	0.0090	0.0010	0.0080	**0.80**	9.00	**0.80**
0.95	0.05	**0.90**	0.0095	0.0005	0.0090	**0.90**	19.00	**0.90**

*AR* Absolute risk, *SAR* Standardized AR, *RR* Relative risk; Boldface: The numerical value is same. SAR' = (1-RR^−1^) / (1 + RR^−1^)

As shown in Table 1, SAR eliminates the influence of the trigger factors on the original AR. The results showed that SAR increased with increasing RR, and SAR' was the same as SAR under the standardized model. An RR of 3.0 yielded a SAR value of 0.50, suggesting that the observed factor could be an effective predictor.

The OR calculated in a case‒control study is similar to the RR calculated in a cohort study under the condition of low probability [12–14]. Therefore, SAR can be obtained using the OR of the case‒control study as follows:

4 $$SAR=\frac{1-{RR}^{-1}}{1+{RR}^{-1}}=\frac{1-{OR}^{-1}}{1+{OR}^{-1}}$$

### Observing the role of genes in schizophrenia

**Fig. 5:**
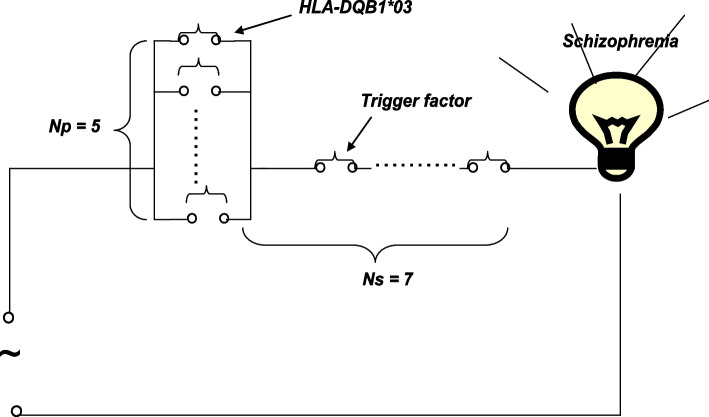
Information visualization for displaying the relationship and strength between HLADQB1*03 and schizophrenia

Our previous study, which had a case‒control design, reported that the frequencies of HLA-DQB1*03 were 0.533 in the SCH group and 0.420 in the control group (*p* = 0.035) [10]. The values of OR, RR, and SAR (OR = RR = 1.58 and SAR = 0.22) were obtained according to Eq. (4), as shown in Table 2; thus, HLA-DQB1*03 is a low-risk gene for SCH. Because SCH affects approximately 1% of the population [7], the results revealed that Ns = 6.6–7 and Np = 4.3–5 according to Eqs. (2) and (3), as shown in Fig. 5.

**Table 2:** Original data for displaying the role of HLA-DQB1*03 in schizophrenia

Case–control design		Incidence in total population	Data visualization	
Disease	Control		Ns	Np
0.53	0.42	1.0%	7	5
OR = 1.58		-	SAR = 0.22

### Role of ageing (in five-year age groups) with death

**Fig. 6:**
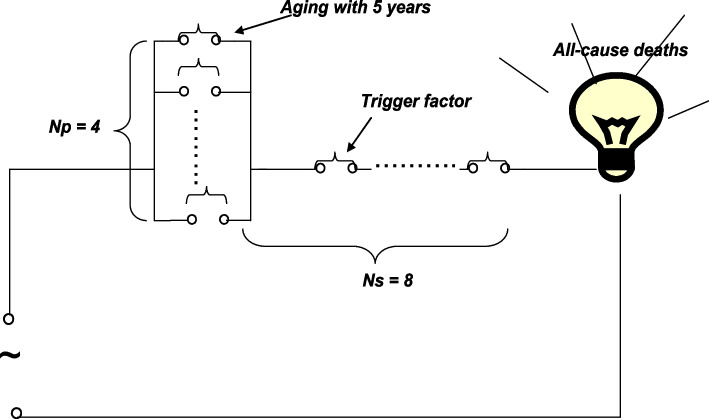
Information visualization for displaying the relationship and strength between aging with 5 years and all-cause death

Age-stratified (age groups: 60–64 years and 65–69 years) all-cause mortality in the monitored population in China in 2011 are listed in Table 3. The role of ageing (as a categorical variable divided into 5-year age groups) with all-cause mortality was calculated using Eq. (1) with the 60–64-year age group as the reference. SAR was 0.321, which indicates that the role of ageing in death had a medium effect size. Because the mortality rate was approximately 0.6% of the total population during one year [15], the results revealed that Ns = 3.1–4 and Np = 7.4–8 according to Eqs. (2) and (3), as shown in Fig. 6.

**Table 3:** Age-stratified all-cause deaths in the monitored population in China in 2011

Age groups	Death	Survival	Death rate	SAR
60 ~ 64	4811	747,831	0.006	0.321
65 ~ 69	5309	424,295	0.013

## Discussion

The present study used two models to determine the relationships among causative factors and their contribution towards a binary outcome. The models were based on the parallel-serial connections of switches, where causal factors were simplified as switches. Therefore, the display of data using switch-on, switch-off and the number of switches can reveal patterns and levels of causal factors.

A binary outcome is derived from a trigger factor and several known and unknown causal factors with a sufficiently large SAR (such as SAR > 0.5) accumulated through a parallel- and serial-connection model. Thus, the observer can intuitively understand the interaction between a causal factor and a result in a complex event through information visualization with switch-on and switch-off. The number of switches reveals factor patterns and levels. The number of factors combined in the present model represent the percentage of the contribution of the observed factors to an outcome (the fewer the number of factors in the model, the greater the contribution of the observed factor is). Therefore, the number of factors in the model can be used as the basis for evaluating the contribution of an observed factor in a study using information visualization with the number of switches.

AR indicates intergroup differences in the incidence in cohort studies and represents the mean probability of the incidence resulting from an observed factor. In fact, SAR and AR are equal when the sum of probability of an outcome in two groups is one. Effect size is the ratio of a result explained by an observed factor [16]; therefore, SAR is also the effect size for positive outcomes under the standardized model. The effect size of SAR was simulated by a standardized parallel-connection model in the present study. According to the standardized parallel-connection model used to evaluate the relationships and the effect size of an observed factor considering trigger factors, SAR values range from 0 to 0.24 for low effect sizes or weakly significant factors (which could be understood as one of more four factors that may play a role in disease incidence under the standard model), from 0.25 to 0.49 for medium effect sizes (which imply that one of two or three factors may play a role in disease incidence), and over 0.50 for high effect sizes (which imply that mainly one factor plays a role in disease incidence). A variable may be a trigger factor when SAR is close to 1.0.

A measured outcome can be considered to have a 100% probability of occurrence without considering the group of the dependent variable. The coefficient of determination (denoted by R^2^) derived from the measured outcome is interpreted as the proportion of the variance in the dependent variable [16]. Thus, SAR could be comparable to R^2^. The guidelines for the interpretation of R^2^ could be used as references for SAR [17–19]. However, all such criteria are arbitrary in some ways. The suitability of SAR for the above intensity assessment is based on the standardized parallel-connection model in the present study. The detailed meaning of SAR in a particular field will vary according to real-life applications.

SAR values > 0.50 are proposed to serve as an effective predictor (implying that less than two factors play a role in an outcome under the parallel model). The results revealed that approximately seven effective predictors are needed to accurately predict SCH (Fig. 5). The SAR value of the HLADQB1*03 gene was 0.22 (indicating a small effect size on SCH), implying that genes are not predictors of SCH. In fact, it is difficult to find seven predictors with an SAR value > 0.5 for genes or other causal factors for SCH.

Analyses of actual data also revealed that the SAR value of ageing (as a categorical variable divided into two 5-year age groups) in death was 0.321 (a medium effect size), implying that other risk factors could also play an important role in death when trigger factors exist. This system of data visualization represents a new avenue for analysing low-probability events and is useful for understanding complicated relationships between observed factors and disease incidence using common sense to make rational decisions.

A significant difference does not indicate the strength of the effect of an observed factor on the outcome. Putative factors with SAR values < 0.25, such as the gene HLADQB1*03 for SCH, may not require an in-depth investigation even if group comparisons are statistically significant. In addition, SAR values > 0.25 represent a substantial effect size and require further investigation. This system of data visualization highlights the importance of identifying trigger factors for studying low-probability events with binary outcomes. A trigger factor means that the frequency of this factor reaches 100% in the case group of a case‒control study (i.e., in the presence of the trigger factor, all cases exhibit the disease) or that the incidence of disease is zero in the nonexposure group of a cohort study. However, it is difficult to find a trigger factor.

Cohort studies examine correlations between diseases and their associated factors. Using a putative risk factor as an exposure variable, exposed and unexposed subjects are observed until they develop the outcome of interest. Cohort studies are thought to yield robust scientific evidence [20–22]. The expectation is that the incidence in the exposure group is 1 or close to 1 despite being a low-probability event. However, a very low incidence of morbidity in the exposure group of a cohort study is commonly observed for a low-probability event. This is partly because of the trigger factor. AR represents the mean probability of incidence for an observed factor and the proportion of the outcome that may be predicted using the observed factor. AR may be incorrect if trigger factors are present. SAR is the standardized AR under the standardized model, as shown in Table 1. More importantly, SAR eliminates the effect of the trigger factor on AR. Therefore, the use of SAR is suggested when evaluating the effect of a factor on disease outcome.

RR is a ratio risk rather than a proportion of the outcome explained by the observed factor. In the present study, the relationship between AR and RR was obtained under the standardized model, as shown in Table 1. Thus, SAR can be obtained using OR values derived from case‒control studies. Case‒control studies can be used to examine the factors of rare diseases. This method is less costly, has a shorter duration and is often the only practical choice [23–25]. Thus, SAR could provide a new pathway for observational research to increase efficiency and efficacy.

Case‒control studies are considered unreliable in the hierarchy of evidence [20–22, 26]. However, the present study showed that case‒control designs were not affected by trigger factors; therefore, case‒control designs could be better than cohort designs. In other words, when the probability of an outcome is low, the frequency of a causal factor in the disease group cannot be very low; therefore, it is easy to obtain accurate data.

When the probability of outcome in the nonexposed group is very low or zero, SAR is close to one, which affects the corresponding results. Therefore, a sufficient sample size is very important when obtaining accurate data to calculate SAR. Case‒control studies may be a realistic choice. When the probability of outcome in the population is not very low, OR does not correspond to RR and could impact the intensity evaluation; therefore, it is suggested to obtain RR using a definite relationship between cohort outcomes and those from the case‒control study for calculating SAR [27].

Another example is the analysis of genetic associations, which have been successfully used to map genes but are clinically inaccurate, partly because of overestimations of the effect of an observed factor on the outcome using RR and overlooking of trigger factors and other factors within the parallel-serial model. Therefore, most results derived from cohort designs or case–control designs should be corrected by SAR to elucidate the actual effect of the observed factor on the outcome in order to find more effective predictor; these parallel-serial models could yield new discoveries.

It should be noted that SAR is not the same as a diagnostic effect. The diagnostic effect considers consistent rates, both observed consistent and predicted consistent. For an indicator with a small SAR, neither consistent rate is adequately equipped to do both at the same time. A comprehensive index of biomarkers, based on our previous work, is recommended for evaluating effects of diagnosis [28].

SAR also differs from risk in toxicology or epidemiology. A risk assessment should consider the interference of trigger factors and other confounding factors on the outcome. RR is more commonly used to evaluate risk than AR, but the present study has shown that RR may not accurately reflect trigger factors, which is an indirect evaluation of the level of the risk factor through confounding factors. An angle compared index with the hybrid of changes in the ratio and amplitude, our previous work, is recommended for evaluating risk level [29].

## Conclusion

In conclusion, it is reasonable to assume the effect of trigger factors and observed factors accumulate in accordance with the parallel-serial connection model for binary outcomes. SAR may allow better evaluation of the effect size of a factor on complex events by eliminating trigger factors. A system of data visualization based on the parallel-serial connection model could increase our understanding of effect size in measures of association using new concepts. The efficiency and efficacy of observational research could be increased if we were able to clarify how multiple factors can be related to a result in a pragmatic manner based on parallel-serial connection models.

## Data Availability

The datasets used and analysed during the current study available from the corresponding author on reasonable request.

## Abbreviations

AR: Absolute risk
m: Morbidity in the total population
Np: Number of observed factors based on parallel model
Ns: Number of predictive factors based on serial model
OR: Odds ratios
RR: Relative risk
SAR: Standardized absolute risk
SCH: Schizophrenia SCH
